# Announcement of two new Main Editors for *Journal of Synchrotron Radiation*

**DOI:** 10.1107/S1600577520014083

**Published:** 2020-11-01

**Authors:** Editorial Office

**Affiliations:** a IUCr, 5 Abbey Square, Chester, CH1 2HU, United Kingdom

**Keywords:** *Journal of Synchrotron Radiation*

## Abstract

Two new Main Editors of *JSR* are announced.

The entire *Journal of Synchrotron Radiation* (*JSR*) editorial team would like to take this opportunity to thank Professor Dr Ilme Schlichting (Max Planck Institute, Heidelberg, Germany) for her many years of service as a Main Editor of *JSR*, and wish her well as she steps down at this time.

Following our advertisement for a new *JSR* Main Editor earlier in the summer, together with other discussions, we are pleased to announce the appointment of two new *JSR* Main Editors:

Dr Dibyendu Bhattacharyya – Head, Synchrotron Science and Multilayer Physics Section, Atomic and Molecular Physics Division, Bhabha Atomic Research Centre, Mumbai, India, and also Professor at Homi Bhabha National Institute, Mumbai, India.
Professor Dr Kristina Kvashnina – ERC group Head at the European Synchrotron – ESRF, Grenoble, France, at the beamline supported by Helmholtz-Zentrum Dresden-Rossendorf, Germany; also Professor of Chemistry, Moscow State University, Russia.

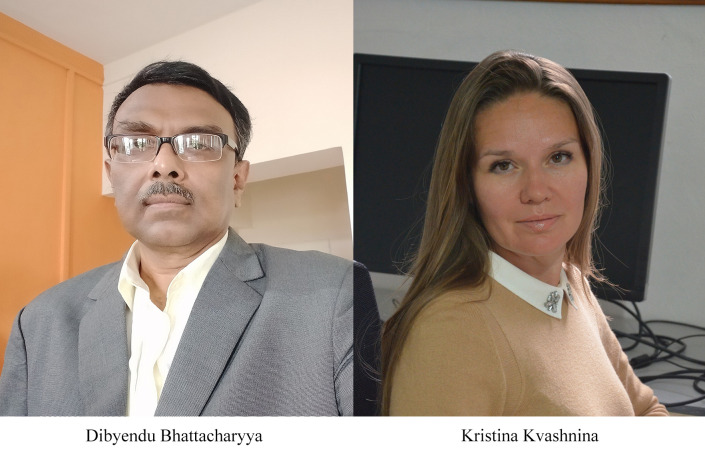


While not previously associated with IUCr journals as such, Dibyendu is actively involved in the IUCr Commission on XAFS, and has published several papers in *JSR*. He will bring in a new regional perspective, and we hope more papers from India as India continues to develop its own major facilities.

Kristina is an existing Co-editor of *JSR*. By being an active committee member of the review panels at several synchrotron and free-electron laser facilities across different countries, she is comfortable with the open access publishing environment, and she will bring valuable insights for guiding *JSR* into the fully open-access regime.

Dibyendu and Kristina will work with *JSR*’s other Main Editors, Professor Yoshiyuki Amemiya (University of Tokyo, Japan) and Professor Ingolf Lindau (SLAC/Stanford University, USA), and of course with the entire *JSR* Editorial Board.

More information to introduce our new Main Editors will be provided in the next issue and in the *IUCr Newsletter*.

